# Resource demands reduce partner discrimination in Himba women

**DOI:** 10.1017/ehs.2020.43

**Published:** 2020-08-24

**Authors:** Sean P. Prall, Brooke A. Scelza

**Affiliations:** 1 Department of Anthropology, University of Missouri, Columbia, MO, USA; 2 Department of Anthropology, University of California, Los Angeles, CA, USA

**Keywords:** mate choice, food insecurity, transactional sex

## Abstract

Where autonomy for partner choice is high, partner preferences may be shaped by both social and ecological conditions. In particular, women's access to resources can influence both the type and number of partnerships she engages in. However, most existing data linking resources and partner choice rely on either priming effects or large demographic databases, rather than preferences for specific individuals. Here we leverage a combination of demographic data, food insecurity scores and trait and partner preference ratings to determine whether resource security modulates partner preferences among Himba pastoralists. We find that while food insecurity alone has a weak effect on women's openness to new partners, the interaction of food insecurity and number of dependent children strongly predicts women's openness to potential partners. Further, we show that women who have more dependants have stronger preferences for wealthy and influential men. An alternative hypothesis derived from mating-market dynamics, that female desirability affects female preferences, had no effect. Our data show that women who face greater resource constraints are less discriminating in the number of partners they are open to, and have stronger preferences for resource-related traits. These findings highlight the importance of ecological signals in explaining the plasticity of mate preferences.

## Graphical abstract

**Figure:**
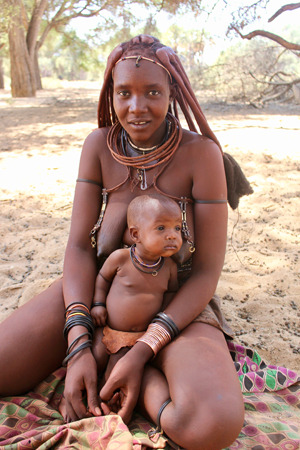

**Media summary:** Cues of resource scarcity, but not relative mate value, shape female preferences in Himba women.

## Introduction

A major tenet of sexual selection theory is that women will exhibit preferences for men with greater resource acquisition potential (Buss, 1989; Daly & Wilson, 1983; Halliday, 1983). Such preferences have been demonstrated in both experimental and naturalistic studies across a number of populations (Bereczkei, Voros, Gal, & Bernath, 1997; Buss, 1989; Buss & Schmitt, 1993; Waynforth & Dunbar, 1995). However, cues of resource potential are just some of the many traits that women have been shown to prefer. Personality characteristics like a sense of humor and kindness, physical attractiveness and indicators of good parenting are all routinely highly ranked by women in mate preference studies conducted around the world (Buss, 1989; Koster, 2011; Marlowe, 2004; Pillsworth, 2008; Schacht & Grote, 2015). In addition, preferences have been shown to be plastic and responsive to changing conditions (Lee & Zietsch, 2011; Lu, Zhu, & Chang, 2015; Scelza & Prall, 2018). The combination of these two factors, the diversity and the plasticity in preferences, raises important questions about how socioecological conditions shape partnership dynamics.

### Resource constraints and female choice

Given that female preferences are often linked to resources, women should be particularly sensitive to signals of resource scarcity. As scarcity increases, women may shift their preferences, placing more emphasis on indicators of wealth and resource potential over other traits. Studies that use psychological priming tasks support this notion, indicating that signals of resource scarcity fundamentally shift female preferences. Priming resource scarcity resulted in increased preference for ‘good-dad’ traits over ‘good-genes’ traits (Lee & Zietsch, 2011). Other studies have shown that priming resource scarcity can change how women perceive male characteristics. Watkins, DeBruine, Little, Feinberg, and Jones (2012) show that when scarcity is primed, women are more likely to equate masculinity with dominance, whereas primes for pathogen stress lead to women linking masculinity with attractiveness. These studies show that woman's preferences are susceptible to external conditions, and that they respond in ecologically relevant ways.

Food insecurity in particular may be a salient driver of partner preferences. Measures of food insecurity include reported access to food, but also worries and concerns about not having enough food (Jones, Ngure, Pelto, & Young, 2013), and as such are believed to be reliable indicators of perceptions of resource scarcity. This scarcity has been linked to a number of indicators of high-risk sexual behavior. In Uganda, food insecurity was associated with an increase in transactional sex, a reduction in condom use and a higher likelihood of staying in violent relationships (Miller et al., 2011). Similarly, food insecurity was associated with reduced condom use, more unsafe sex and multiple sexual partners in marginalized HIV-infected populations (Cluver, Orkin, Boyes, Gardner, & Meinck, 2011; Pascoe et al., 2015a; Shannon et al., 2011; Vogenthaler et al., 2013). As opposed to the priming studies, which show shifts in preferences for different male traits, these studies show resource scarcity linked with openness to a greater number of partners, and riskier behaviors with those partners.

The role of female choice in these circumstances is hotly debated (Stoebenau, Heise, Wamoyi, & Bobrova, 2016). Within the transactional sex literature some argue that women are coerced into sex-for-resources relationships owing to economic marginalization and patriarchal social structures (Cluver et al., 2011; Dunkle et al., 2004; McCleary-Sills, Douglas, Rwehumbiza, Hamisi, & Mabala, 2013; Wojcicki, 2002). Others argue for more female agency, positing that women actively engage in negotiations, marketing sexual favours in exchange for needed or desired goods (Silberschmidt & Rasch, 2001; Wamoyi, Wight, Plummer, Mshana, & Ross, 2010). There is also debate about how much factors like food security and poverty influence participation in transactional sex relationships. Some studies show clear links between engaging in transactional sex and material deprivation, a lack of adequate food, housing or health care (Greif, 2012; Kamndaya, Thomas, Vearey, Sartorius, & Kazembe, 2014; Nzyuko et al., 1997). However, these transfers are not just about alleviating immediate need; they are also linked to signals of romantic commitment and social dynamics (Poulin, 2007; Verheijen, 2011) as well as the acquisition of consumer and luxury goods (Kamndaya, Vearey, Thomas, Kabiru, & Kazembe, 2016; Leclerc-Madlala, 2003; Masvawure, 2010).

### Mating market dynamics

Mating decisions exist at the level of the individual, but they also operate within the larger social sphere. This means that partner choice is influenced by the availability of potential mates, the relative quality of potential mates and the competitive interactions between mate-seekers (Noë & Hammerstein, 1995). Women who are highly desired, owing to some intrinsic or extrinsic quality, can leverage more influence in the mating market than women who are less desired can. For example, individuals who perceive themselves to be more desirable have been shown to have more selective partner preferences (Buston & Emlen, 2003; Fales et al., 2016; Lee, Loewenstein, Ariely, Hong, & Young, 2008). Other studies have shown that women and men calibrate their mating aspirations in the face of experimentally derived rejections or acceptances (Kavanagh, Robins, & Ellis, 2010; Reeve, Kelly, & Welling, 2017). Assortative mating studies similarly show that people tend to pair off with partners of similar quality (Buston & Emlen, 2003). This can create a confounding effect on the relationship between resource scarcity and partner choice. If women with greater resource needs are less desirable on the mating market, they may be open to a wider selection of potential mates because they need to be to find a partner, not because their resource insecurity is leading them to be open to more partners.

### Aims of the current study

Current work within the social sciences on partner preferences and their relation to resource scarcity is focused on two areas: (1) psychological experiments conducted in industrialized countries; and (2) the nature and dynamics of ‘transactional sex’. The former relies heavily on hypotheticals and tends to utilize populations from wealthy nations where resource scarcity is limited (Lee & Zietsch, 2011; Watkins et al., 2012). The latter is a mix of large-scale demographic studies that lack localized specificity (Pascoe et al., 2015b; Shannon et al., 2011) and qualitative ethnographic studies that provide important context but do not include quantitative measures of scarcity or preference (Miller et al., 2011; Tawfik & Watkins, 2007). Little work has been done in communities where extra-marital partnerships are normative and common for all individuals regardless of health or social status. To better understand how resource constraints shift mating preferences, we focus on a population of pastoralists where concurrency is common and relatively de-stigmatized. Furthermore, in opposition to studies that use exclusively psychological tasks, we use a novel photographic rating task, where participants rate and rank known individuals in their community. Based on the literature outlined here, we predict that women with greater resource needs will be open to more partners. Further, we predict that wealthier men, and men that possess traits related to resource acquisition (influential, hardworking) and resource sharing (generosity), will be more highly preferred by women under resource stress. We compare these predictions with a mating market model, which predicts that preferences will be shaped by a woman's relative bargaining power.

## Methods

### Study population

This study was conducted as part of a larger project on partner choice and family dynamics conducted in northwestern Namibia. The study focuses on the members of ~45 Himba households living in the Omuhonga basin, many of whom have been part of this study since 2010. Himba are semi-nomadic pastoralists, although in Omuhonga women's gardens, primarily of maize, are important supplements to the milk and meat in their diet (Bollig, 2010). Access to the market economy is still relatively limited, with the exception of livestock sales and pension payments, but increasing access to secondary education is indicative of a shift towards increasing reliance on the cash economy. Both men and women can accumulate livestock wealth, but only men inherit substantial livestock herds. Women tend to have only a few sheep and goats, although older women who are the head of their household may accumulate a large number of small stock. Electricity and running water are still absent from the community. However, most adults now have cell phones and a small number of men have vehicles.

Households are typically polygynous, extended families, ranging in size from eight to 25 individuals. Marriages are all arranged, although in many cases couples ‘choose each other’ and then get formal permission to marry from their families. Polygyny in this population co-occurs with a high degree of female autonomy and women are able to travel without their husbands for extended periods (Scelza, 2011b, 2015). Divorce is frequent, and can be initiated by either spouse. Additionally, concurrent partnerships are common for men and women, married and unmarried, and numerous cultural norms protect the maintenance of these informal unions (Scelza, 2013; Scelza et al., 2020b; Scelza & Prall, 2018). Informal partnerships range from brief encounters to lengthy relationships, some of which span the births of multiple children, and some which ultimately end in marriage. Informal partnerships occur with both unmarried individuals and those who are married to someone else, although the latter is treated with much more secrecy than the former. This practice results in a high rate of extra-pair paternity, with 48% of children born to married couples fathered by someone other than the husband (Scelza et al., 2020b). Strong social norms encourage men to provide for all marital children, despite suspected paternity, and evidence of biased paternal care is limited (Prall & Scelza, 2020; Scelza et al. 2020a). The regularity of non-marital relationships and the openness with which Himba men and women speak about them make this an ideal population to study partnership dynamics. Additional ethnographic information can be found in Bollig (2010).

### Data collection

Data for this study was collected between 2016 and 2018, with some longitudinal data collected as far back as 2010. As part of a larger study on health, reproduction and demography, Himba men and women were recruited opportunistically from the study area when conducting compound visits. Participants underwent standard demographic interviews, which included reproductive histories, martial status and number of livestock owned. Age was calculated using the traditional year-name system used by this population (see Scelza, 2011a for details on this method). We calculated number of dependants as number of children under 10 years of age, and exclude children being foster raised. Children younger than 10, unlike older children, contribute little to the household economy, and are more burdensome to care for. Older children are more valuable for their labour, contribute substantially to the household through domestic tasks, and are viewed as an essential workforce for a successful household. In assessing the impact of number of dependants as described here, on partner preference, we only use data from women who had at least one dependent child.

Women completed a five-item three-response food insecurity questionnaire, the household hunger scale, modified from Deitcher, Ballard, Swindale and Coates (2010) (described in the Supplementary Materials). Responses were coded into a single numeric variable. For a subset of participants, food insecurity scores were reported across multiple years. For these individuals, scores were averaged and rounded to a whole number. To correct for effects of age and marital status on food insecurity outcomes, standardized residuals (and error) were generated from a truncated Poisson regression, and used as predictors in the main model.

To estimate cattle wealth, longitudinal livestock counts from all men between 2010 and 2018 were converted to log-transformed tropical livestock units using standard conversion metrics (Bollig, 2010). These values were then fit to a Gaussian regression with varying intercepts by participant, to predict overall wealth levels for 79 men.

In addition to the life history interviews described above, a subset of participants were recruited to complete a rating and partner preference task. First participants were shown a series of digital headshots of individuals in the community on a tablet computer (Figure S1). Participants were asked, for each photo, if that person was a certain trait (generous, hardworking, attractive, influential), with a binary yes/no response for each. After the participant had completed 10–20 ratings on a single trait, they moved to the a different trait and rated an additional set of photos. Photos were displayed at random in each iteration. For these analyses, we only use female status ratings of opposite-sex individuals, since the outcome variable is female preference ratings. This task resulted in 7159 trait ratings across four different traits by 92 women. Of total cases, 54.4, 59.4, 28.8 and 35.6% of ratings for generous, hardworking, attractive and influential (respectively) were in the affirmative. Ratee trait probabilities were calculated by fitting a multilevel model, with varying intercepts for rater and ratee.

In the second task, participants were shown a series of digital photos of opposite-sex individuals, and were asked how desirable that individual was to be in a relationship with (Figure S2). Participants answered this question with a four-item Likert scale (none/low/medium/high). Participants rated as many individuals as possible in this task, up to 100 individuals. For this analysis, we exclude ratings where women were more than 10 years older than the man in question. This task resulted in a 6972 female preference ratings of opposite-sex individuals, used as the outcome variable for the main models described here. Since not all measures (food insecurity, dependants) were collected or known for all individuals, sample size varies with inclusion of predictors, with the null model consisting of 6972 ratings from 96 women, to the final interaction model consisting of 3992 ratings from 56 women (Table S1). To assess female quality, varying intercept coefficients (with standard deviation of the posterior as an estimate of measurement error) were derived from models of men predicting the desirability of women (3851 ratings of 173 women by 49 men).

### Data analysis

An ordered logit regression with varying intercepts for rater and ratee and fixed-effect predictors was used to estimate female preference. These predictors included standardized age, standardized age difference between rater and ratee, centred number of dependants and standardized food insecurity score. To assess how male traits might influence female preference ratings, additional models included standardized male trait probabilities and two-way interaction effects for dependants and food insecurity. All models included regularizing priors for predictors (*β* ≈ *Normal* [0,1]), and variance parameters (*σ* ≈ *Exponential* [1]). Models were fit to *RStan* (Stan Development Team, 2019) using the *brms* package (Bürkner, 2017), and convergence assessed via 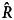 scores. Some participants had missing information (age and age difference), so these values were imputed during model fitting using the *mi()* command as part of the *brms* package, with the same priors as described above. Likewise, when possible, measurement error was included in predictors using the *me()* function. Full model descriptions, plotted raw data and plotted posterior predictions are shown in the Supplementary Materials. Where presented below, model results represent the posterior mean and 95% high-density predictive intervals (HDI), as well as the percentage of the posterior distribution above or below zero.

## Results

### Food insecurity and partner preference

Women were highly discriminatory in their preference ratings (Figure S5). In the null model, of 6972 ratings, 72.70% of ratings were the lowest ‘none’ rating, while only 4.80% of ratings were the highest rating. Rater age increased the probability of positive ratings (*β* = 0.96, 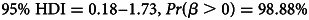), but not when combined with number of dependants or food insecurity (Figure S12). Age difference between rater and ratee had little impact on preference ratings.

**Figure 1.**
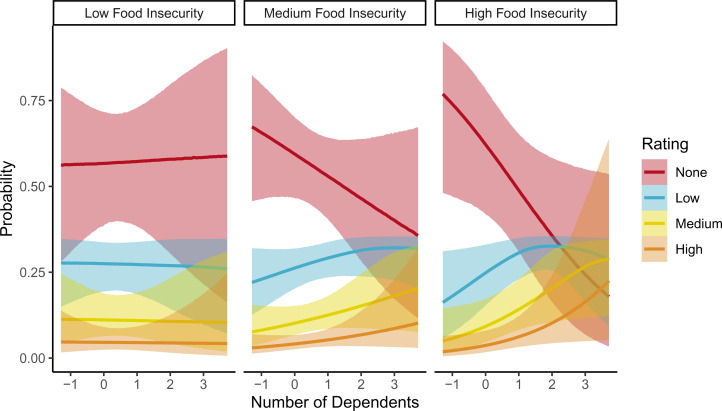
Posterior predictions for interaction effect of number of dependants and food insecurity residuals on reported mate preferences. Posterior medians and 95% credible intervals for each rating category shown.

Number of dependants increased the probability of positive ratings with (*β* = 0.27, 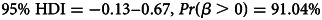, Figure S13) and without (*β* = 0.3, 95% HDI = 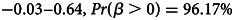, Figure S9) food insecurity residuals and the interaction parameter, so that each additional dependant resulted in a 5.70% lower probability of giving any individual man the lowest rating for partner desirability. However food insecurity alone only modestly impacted rating probabilities (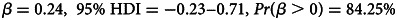, Figure S10). The addition of number of dependants to food insecurity residuals eliminated the impact of food insecurity (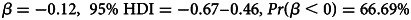), but the interaction parameter between food insecurity residuals and number of dependants was positive and meaningful (*β* = 0.29, 95% HDI = 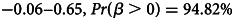), so that in women with high, but not low, food insecurity, a higher number of dependants increases the probability of positive ratings (Figure 1). As an example, a woman with lower than average food insecurity and number of dependants is predicted to have a 18.80% higher probability of giving a lowest rating for partner desirability, relative to a woman with higher than average food insecurity and dependants. In this model, for a woman with average food insecurity, each additional dependant results in a 5.70% decrease in the probability of giving the lowest rating for partner desirability.

### Male wealth and partner preference

Male wealth increased the probability of positive ratings (Figure S17), both in the model including only fixed effect predictors for age, but also in models with food insecurity, number of dependants and interactions with wealth (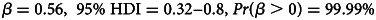). Likewise, when wealth and interactions are included in the model, the interaction term between dependants and food insecurity remains positive and meaningful (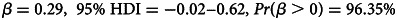). Posterior predictions of relationships between food insecurity, dependants and wealth indicate that women with more dependants, but not higher food insecurity, exhibit stronger preferences for wealthier men (Figure S18).

### Male traits and partner preference

**Figure 2.**
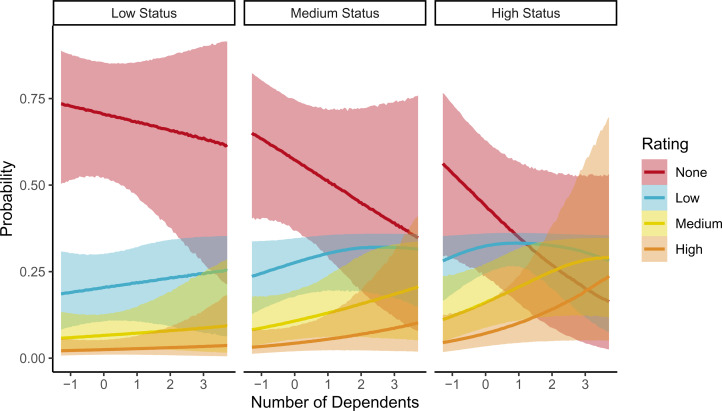
Posterior predictions for interaction effect of number of dependants and status assessments on reported mate preferences. Posterior medians and 95% credible intervals for each rating category shown

We then examined female ratings of male traits in the context of resource scarcity. In these models, the probability of individual men being rated on a trait was centred and used as a fixed effect variable, and each trait run individually with resource traits and interactions. Results indicate that men viewed by women as more influential are more preferred with increasing number of dependants (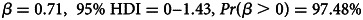, Figure 2), but not food insecurity (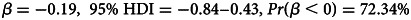, Figure S21). However, attractiveness, generosity and hardworking were generally preferred by women overall, but showed no interaction with food insecurity or number of dependants (Figures S19–S22). The relationship between generosity and wealth was also examined, as previous research showed that generosity was a particularly salient trait in relation to resource transfers (Scelza & Prall, 2018). While wealth remained a meaningful predictor of preference, the interaction between wealth and generosity did not influence preference (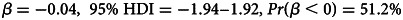).

### Female quality and partner preference

Finally, we tested the alternative hypothesis that women who are viewed as lower quality will be open to a wider variety of men, regardless of resource need. Women's value on the mating market was determined by looking at how men rated women as a potential partner. Varying intercept coefficients derived from models of men rating desirability of women were used as fixed effect predictors. Male-assessed female quality had no substantial impact on female preference ratings (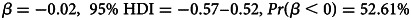, Figure S23).

## Discussion

Male access to resources is believed to be one of the primary factors in female mate choice, and has broad empirical support across taxa. Here, we examine how resource scarcity affects women's openness to new partners, and how the relative importance of resource-related traits changes as resource scarcity increases. Women with more dependants, and women with more dependants who are more food insecure, show attenuated discrimination of potential partners, and are more likely to give any individual male higher preference ratings. We also predicted that other resource related traits, as rated by women in the study community, should be similarly related to female preference with higher resource scarcity predicting greater weighting of these traits. Livestock wealth, as reported by men, was a strong predictor of female preference. However, other male traits which may signal resource acquisition or sharing potential, including being generous and hardworking, showed no relationship to resource need. In other words, while women with more dependants tended to prefer wealthier men, the degree to which a man was viewed as being hardworking, generous or attractive did not differ across women with varyng resource need.

Our data show a positive correlation between resource scarcity and the level of discrimination used in a partner choice task. While definitively demonstrating a causal relationship between scarcity and selectivity is not possible with these data, we did test an alternative hypothesis that this relationship was simply the product of a relationship between resource need and position on the mating market, Our results indicate that relative mate value, as measured by male desirability ratings of women, has no impact on female preferences. Women who were viewed as more desirable by men were not more discriminating in their judgments. Given the large and multifaceted literature on assortative mating, this finding warrants further study. In particular, use of women's assessments of their own desirability (self-perceived mate value), instead of male assessed desirability, may show stronger associations with women's mate choice. Here, we focus on the role that resource scarcity plays in determining preferences, but we recognize that this is just one of many potential pressure points that could be influencing partner choice.

The methods used in this study differ markedly from those that are currently standard in mate choice studies within evolutionary psychology. We rely on individualized demographics and food security ratings, rather than primes of resource scarcity. We also collected preference data for members of the respondents’ own community, people well known to them, rather than standardized images or priming vignettes. This more individualized approach is believed to increase ecological validity in these sorts of tasks (Gervais, 2017). Furthermore, the study was conducted in a community where resource scarcity is particularly salient, as chronic drought and limited access to market goods mean that food insecurity is a common concern. This study therefore provides an important complement to previous findings, which have largely relied on samples from student populations in countries where resource scarcity is less common.

We found that number of dependants was an important predictor of partner preferences, both on its own and in conjunction with food insecurity. In the case of shifting trait preferences, number of dependants was a stronger predictor than food insecurity. It may be because food insecurity is chronically high in this population that food security alone does not produce enough variation to see large effects. Number of dependants represents a longer-term measure of resource stress than food security, which has seasonal components, and which may fluctuate somewhat depending on conditions like the number of recent funerals or ceremonies (where food is more abundant), as well as cultural practices to cope with drought and low food availability (Bollig, 2010).

The results from this study add an interesting complement to the broader literature on transactional sex and risky sexual behavior. Concurrent and sequential partnerships are common among Himba, and not stigmatized in the way they are in many places. The majority of married men and women across age groups have at least one additional partner, and these relationships are often long-lasting, at times spanning several decades (Scelza et al., 2020b; Scelza & Prall, 2018). The transfer of resources is commonly cited as an important component of women's relationships with both their husbands and their lovers. These can range from expectations about provisioning of cash or food to help with an emergency to smaller food gifts and trinkets such as bracelets and mobile airtime. We therefore see the relationship between resource stress and openness to romantic partners to be driven in part by expectations that men can buffer shortfalls, in much the same way that transactional sex operates in other sub-Saharan contexts.

### Limitations

This study uses a novel trait and partner preference trait rating task, where participants rate individuals known to them in the community. In most cases, this is preferable to using self-perceived traits, in that it assesses community perception of individual community members, and as such should be a more accuate representation of individual characteristics. However, this means that these results are difficult to compare with many similar studies that use self-perceived mate value (e.g. Fisher, Cox, Bennett, & Gavric, 2008). Additionally, trait ratings of known individuals may be subject to bias based on personal history, and traits like attractiveness are subject to non-physical influences when assessing known individuals (Kniffin & Wilson, 2004). However, other ratings including being influential and hardworking require knowledge of the individual in question, and the statistical methods make idiosyncratic ratings unlikely to impact results. Complex interactions including marital status of the rater and ratee, interpersonal dynamics and other unknown effects may be at play in the preference ratings. Additionally, in our preference task, women were asked about how much they would like to be in a relationship with a given set of men, but we did not specify whether this would be a marital or non-marital relationship. Since divorce and remarriage are common, as are poygyny and concurrency, partnership status is not a disqualifer of a potential future partner, or partner interest more broadly, but future work will seek to clarify how women best utilize different partner types.

There are other potential explanations for the relationship between resource scarcity and partner preferences that were not directly tested in this study. Some practitioners of life history theory predict that early life stressors (including reduced access to resources) might lead to a faster life history, including being open to a greater number of sexual partners (Simpson, Griskevicius, Kuo, Sung, & Collins, 2012). Because of limitations in the data we had available and questions of ecological validity, we did not test this theory here. Food insecurity in this population is largely a function of access to livestock and maize, both of which are highly dependent on rainfall. In this drought-prone environment fluctuations in wealth are not uncommon, so that a family with plentiful resources one year could after a multiyear drought be suffering greatly. Given this stochasticity, as well as demographic factors like high rates of divorce, remarriage and fosterage, which influence household composition, we do not have a simple measure of early life stress that we could use here as a predictor. Furthermore, in our ethnographic interviews women and men have continually stressed the importance of resource transfers as being an important facet of both formal (marital) and informal romantic relationships. Therefore, we focused here on how current measures of need might impact partner choice.

## Conclusions

Consideration of an evolutionary psychology framework on mate choice, integrated with locally relevant cues of ecology and cultural norms, provides a more nuanced understanding of the relationship between partner choice and resource scarcity than either approach could provide on its own. Our findings support the general notion that resource scarcity affects women's preferences, and in expected directions. However, in looking at the effects of two highly relevant cues of scarcity (food security and number of dependants) we reveal some complex relationships. Food security, a more acute measure of scarcity, is not as meaningful a predictor as number of dependants, which both on its own and in combination with high food insecurity was strongly predictive of women's partner preferences. The fact that both choosing a romantic partner and raising a child are long-term endeavours may provide some clues as to why this was the more salient predictor. Future work on how partner preferences are shaped by ecological inputs could expand upon this finding. In addition, cross-cultural work exploring the relevance of different resource cues based on the degree of female dependence on male resources or the extent of paternal investment could further illuminate how the primary relationship between resources and mate choice manifests in variable settings.

## Data Availability

The datasets generated as part of this study are not publicly available in order to preserve confidentially and the anonymity of members of the study community. Reasonable requests to view data can be sent to the corresponding author.

## Supplementary material

For supplementary material accompanying this paper visit http://dx.doi.org/10.1017/ehs.2020.43. click here to view supplementary material
