# Supplementary material for: Resource demands reduce partner discrimination in Himba women
Source: Evol Hum Sci. 2020 Aug 24;2:e45. doi: 10.1017/ehs.2020.43 (PMC10427436; doi:10.1017/ehs.2020.43)
Supplement: Supplementary file 1 [file S2513843X20000432sup001.pdf]

Supplementary Materials | Resource demands reduce partner discrimination in Himba women

Evolutionary Human Sciences

Sean Prall & Brooke Scelza

2020-07-22

Additional methodological description - Food insecurity, trait, and preference ratings

A food insecurity questionnaire, modified from Deitcher et al. (2010), was used. Participants were asked to assess these questions for themselves and their family, over the past month. Responses included never, rarely/sometimes, and often, and responses coded into number (0-2) and summed for a food insecurity score.

- "Did you or any household member have to eat a smaller meal than you felt you needed because there was not enough food?"
- "Did you or any other household member have to eat fewer meals in a day?"
- "Was there never no food (of any kind) to eat in your household?"
- "Did you or any household member go to sleep at night hungry?"
- "Did you or any household member go a whole day and night without having eaten anything?"

In addition to food insecurity and other demographics, participants completed two tasks as described in the manuscript text. In the first prompt, participants were asked to rate individuals on a set of yes/no characteristics. In the second prompt, participants were asked "how much would you want to be in a relationship with this person?" A likert-scale rating of none/low/medium/high was described as potential responses, and translators checked for comprehension prior to beginning the task. For both tasks, a skip option was available, for example if the image showed kin or was unfamiliar to the rater. When rating attractiveness, participants were encouraged to make a yes/no response whether nor not they knew the individual in the photo, but for all other traits they were to skip photos of individuals who they didn't know and couldn't characterize. For the second task, participant were also asked if they had ever had sex with the individual shown, and whether they had ever had a child with the individual shown. An example of both prompts are shown in **Figure S1 & S2** below. An example of a Himba woman and a translator completing one of the tasks is shown in **Figure S3**.

Figure S1 - Task 1 Example Prompt

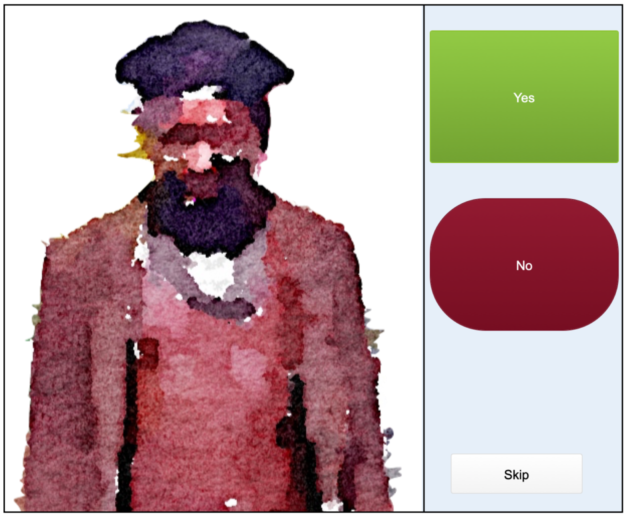

Figure S2 - Task 2 Example Prompt

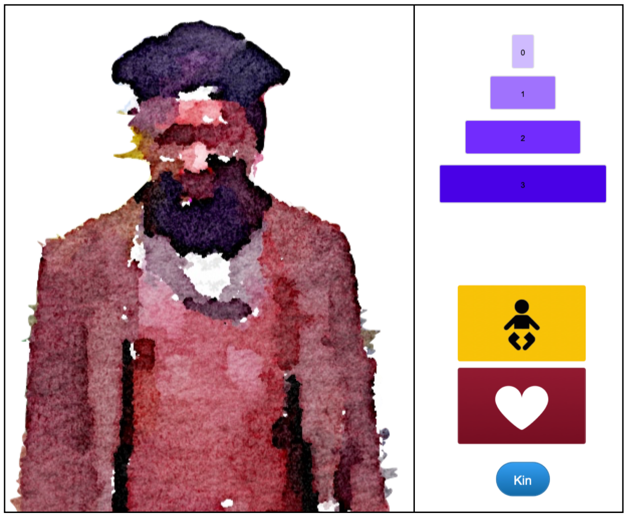

Figure S3 - Example of Himba woman and translator completing rating tasks Used with permission.

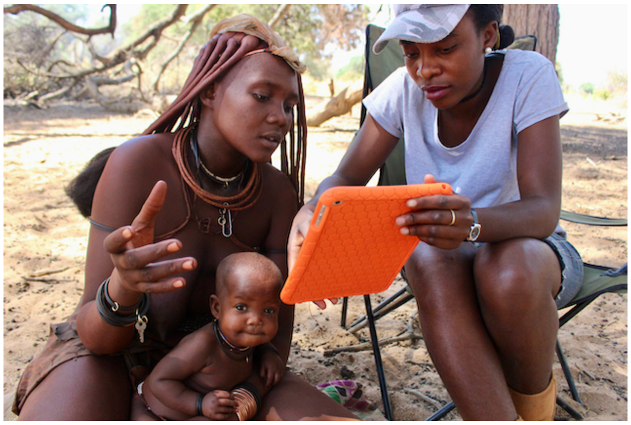

Additional sample description

Models included a maximum of 96 women with relevant predictor information who completed a total of 6972 ratings. Women in this sample had an average of 2.63 (SD = 1.29) dependents that fall within the criteria outlined in the manuscript. Average age for these raters was 29 (SD = 9.31) years and 44% are married. In the 131 men being rated as partners, the average age was 40.21 (SD = 18.52) years. The average age difference between ratee and rater was -15.25 years (SD = 18.46). Figure S4 below shows the distribution of food insecurity scores, and figure S5 illustrates the zero-inflated nature of preference ratings in this sample.

Figure S4 - Distribution of food insecurity scores

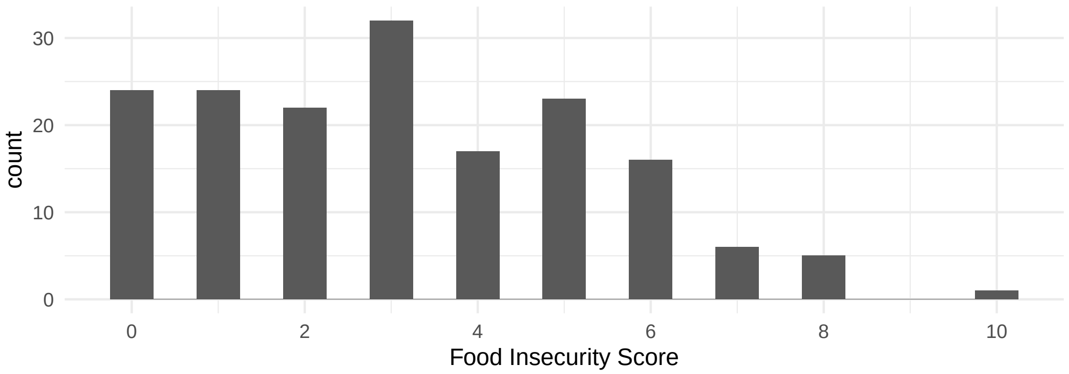

Figure S5 - Distribution of ratings

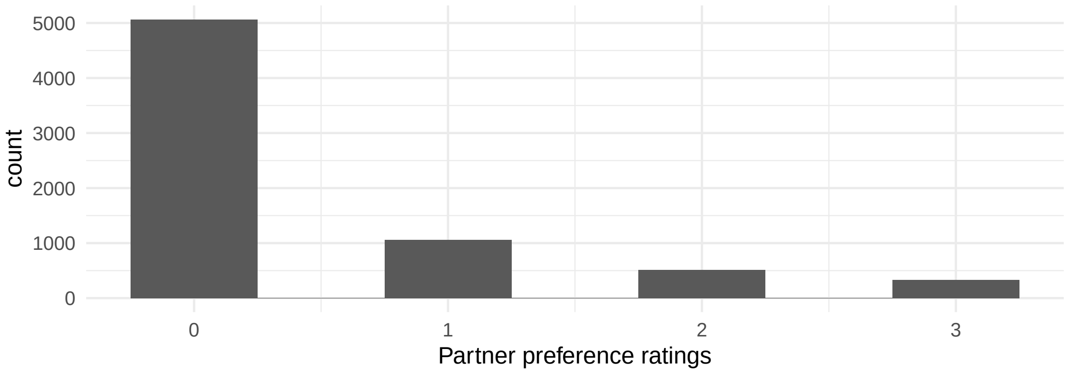

Model descriptions

Modeling food insecurity Standardized residuals and residual error were generated from the following model:

$$T_i \sim Poisson(\lambda_i)$$
$$log(\lambda_i) = \alpha_i + \beta_{Age_i} \cdot Age_i + \beta_{Married_i} \cdot Married_i + \beta_{Interaction_i} \cdot Age_i \cdot Married_i$$

Outcome distribution was defined as truncated at 10, as this is is the maximum number for the food insecurity instrument used in this study.

Modeling trait probabilities To estimate the probability of any individual male being rated a trait, traits probabilities for individual ratees were calculated by fitting the following model, where individual rater and ratees were corrected for using varying intercepts:

$$Trait_i \sim Bernoulli(1, p_i)$$
$$logit(p_i) = \alpha + \alpha_{RateeID[i]} + \alpha_{RaterID[i]}$$

Modeling preference ratings To estimate preference responses models were fitted to an ordered logit regression, with varying intercepts for rater and ratee, as well as fixed effect predictors as shown below as $\beta_{X_i} \cdot X_i$:

$$Rating_i \sim OrderedLogit(\theta, \kappa)$$
$$\theta_i = \alpha_{RateeID[i]} + \alpha_{RaterID[i]} + \beta_{X_i} \cdot X_i ...$$

All analyses were run in R (Team 2019) with the *brms* package (Bürkner 2017). Fixed effect predictors of standardized food insecurity residuals and trait probabilities included error estimates via the *me()* function. Missing values for rater age and age difference were imputed directly in the model using the *mi()* function. All models included regularizing priors for predictors ($\beta \sim Normal[0, 1]$), and variance parameters ($\sigma \sim Exponential[1]$). Other statistical packages used include *bayesplot* (Gabry and Mahr 2019), *tidyverse* (Wickham 2017), *cowplot* (Wilke 2017), *broom* (Robinson and Hayes 2019), *modelr* (Wickham 2020), and *tidybayes* (Kay 2020).

Table 1: Model Descriptions

Models	Coefficients	N.Ratings	N.Ratees	N.Raters
Mod1	Varying intercepts for rater and ratee	6972	131	96
Mod2	Mod1 + Rater age + Age difference	6972	131	96
Mod3	Mod2 + Number dependents	5523	127	75
Mod4	Mod2 + Food insecurity	4746	129	66
Mod5	Mod2 + Dependents + Food insecurity + interaction	3992	127	56

Comparing coefficients across models

Plots compare posterior distributions of coefficients across models. Untransformed posterior median and 95% credible interval shown. Sample sizes between models differ as not all variables are known for all participants.

Figure S6 - Comparison of coefficients

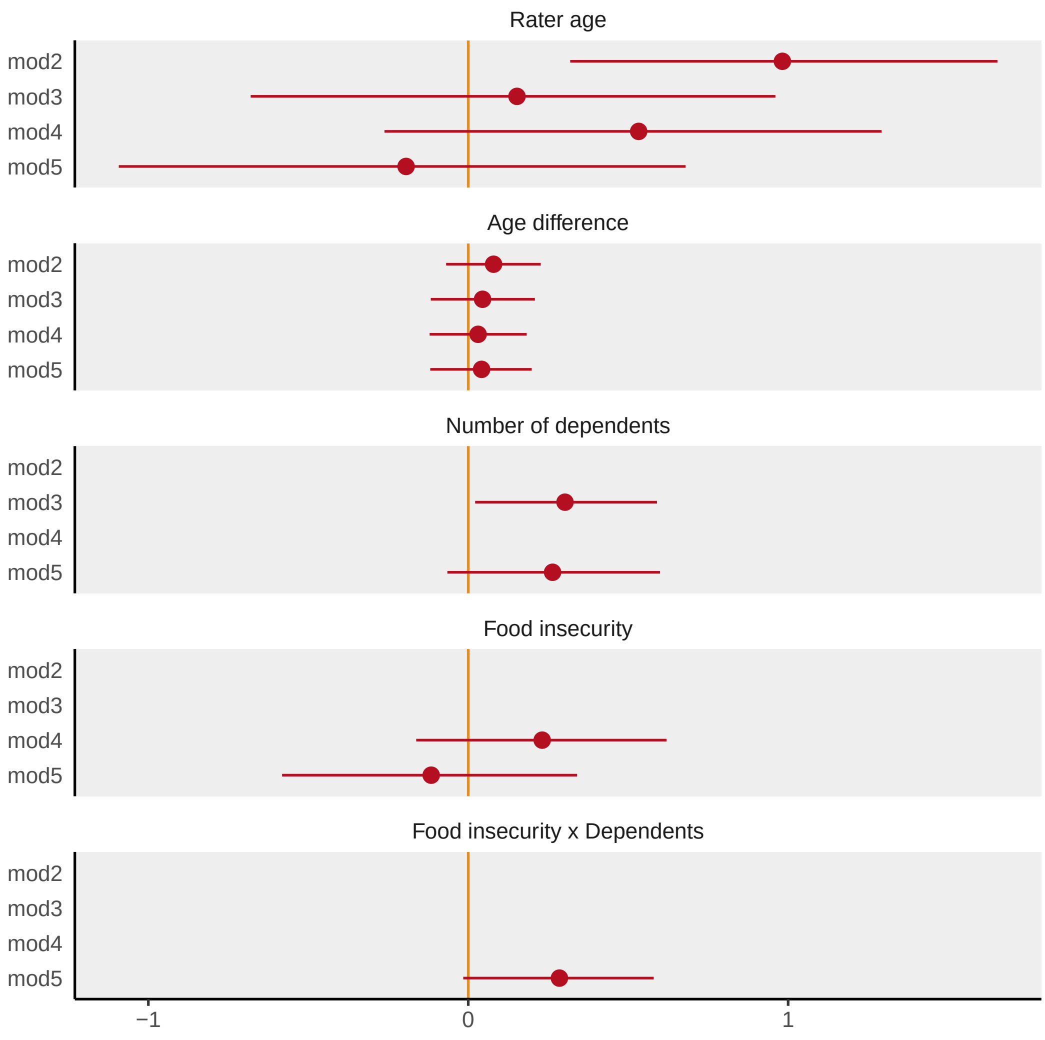

Comparing coefficients across models with complete cases for model 5

Plots compare posterior distributions of coefficients across models, rerun using only complete data for model 5. Untransformed posterior median and 95% credible interval shown.

Figure S7 - Comparison of coefficients - complete cases

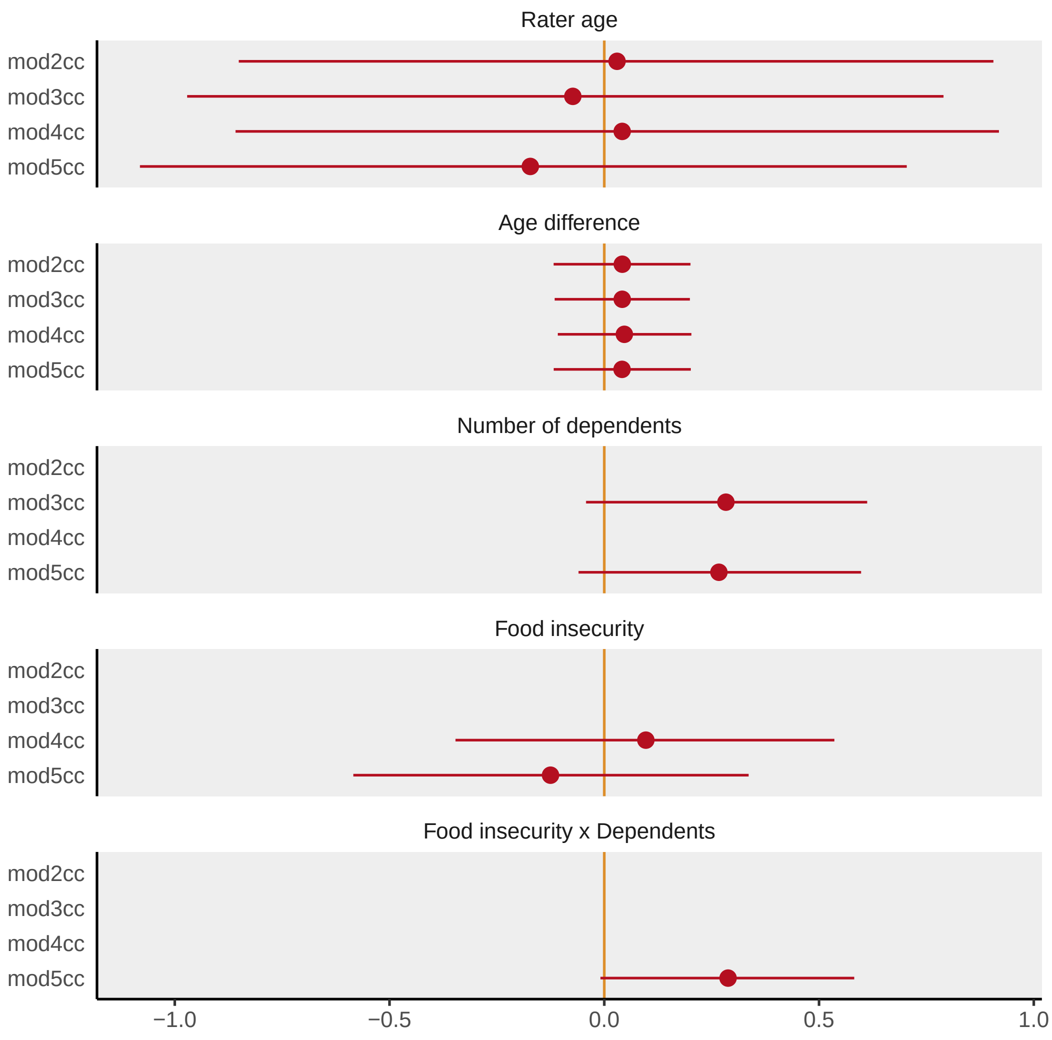

Figure S8 - Comparison of variance estimates across models

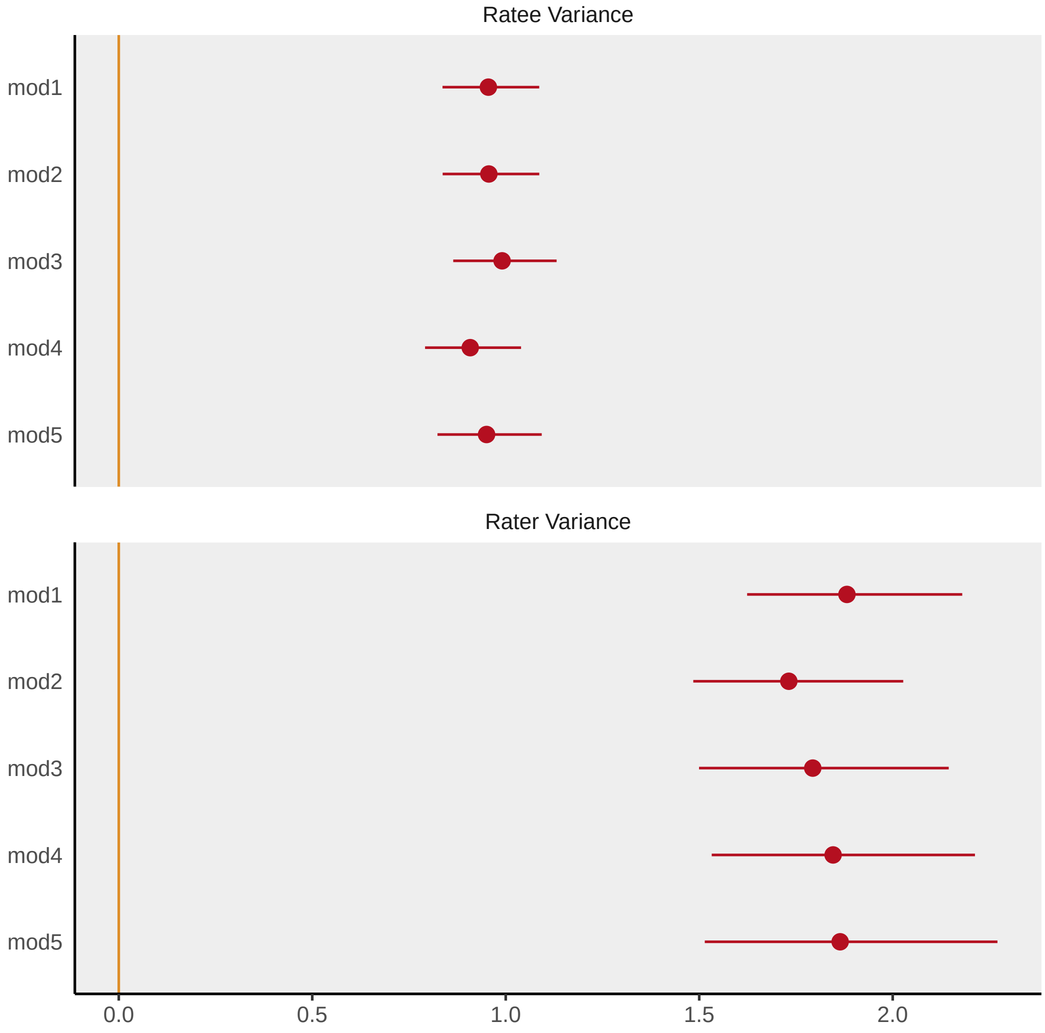

Posterior predictions for number of dependents only from model 3

Posterior predictions of fixed effects parameter for model 3. Raw data on left plots shows jittered responses by predictor. Ordinal predictions in plot on the right show predictions for the probability of each individual rating, with posterior median and 50%, 80%, and 95% credible intervals shown.

Figure S9 - Number of Dependents

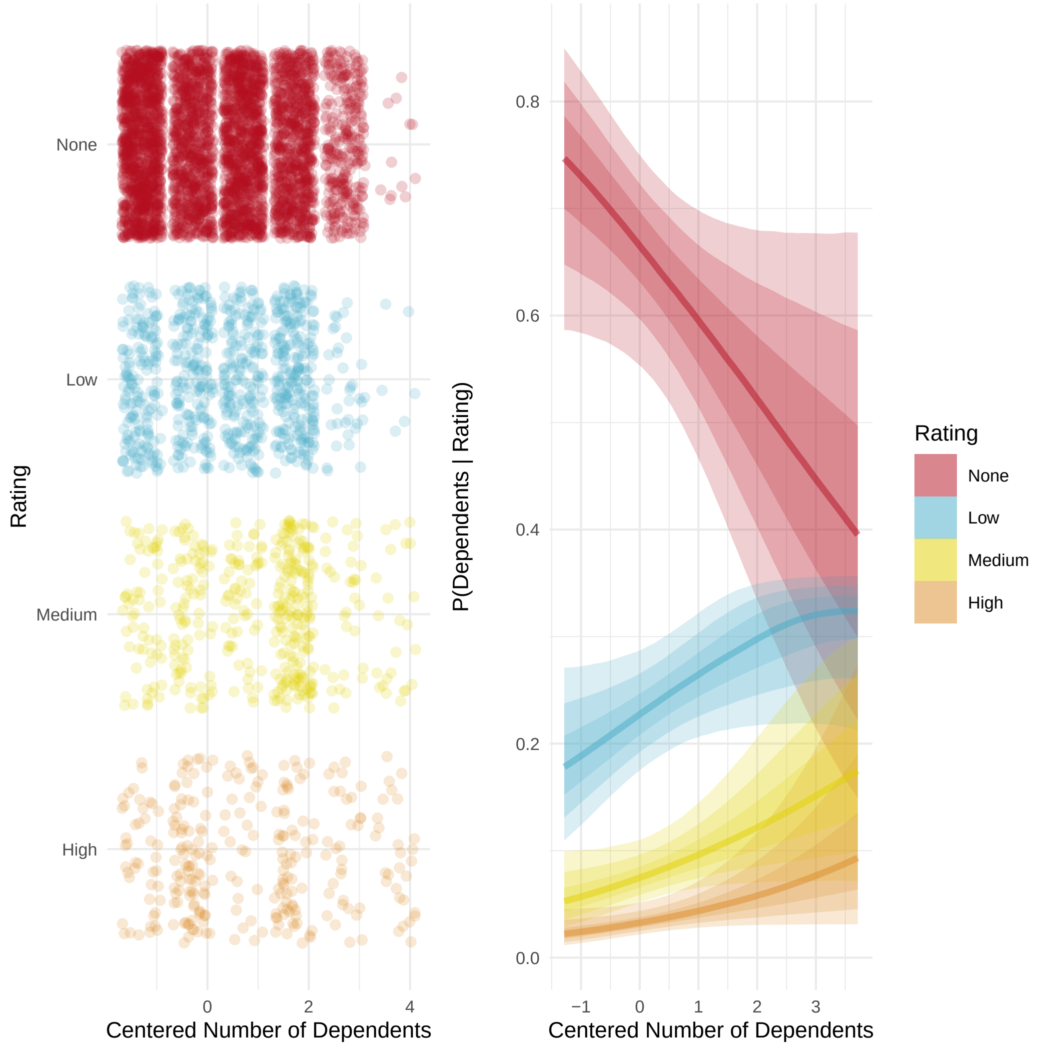

Posterior predictions for food insecurity only from model 4

Posterior predictions of fixed effects parameter for model 4. Raw data on left plots shows jittered responses by predictor. Ordinal predictions in plot on the right show predictions for the probability of each individual rating, with posterior median and 50%, 80%, and 95% credible intervals shown.

Figure S10 - Food Insecurity

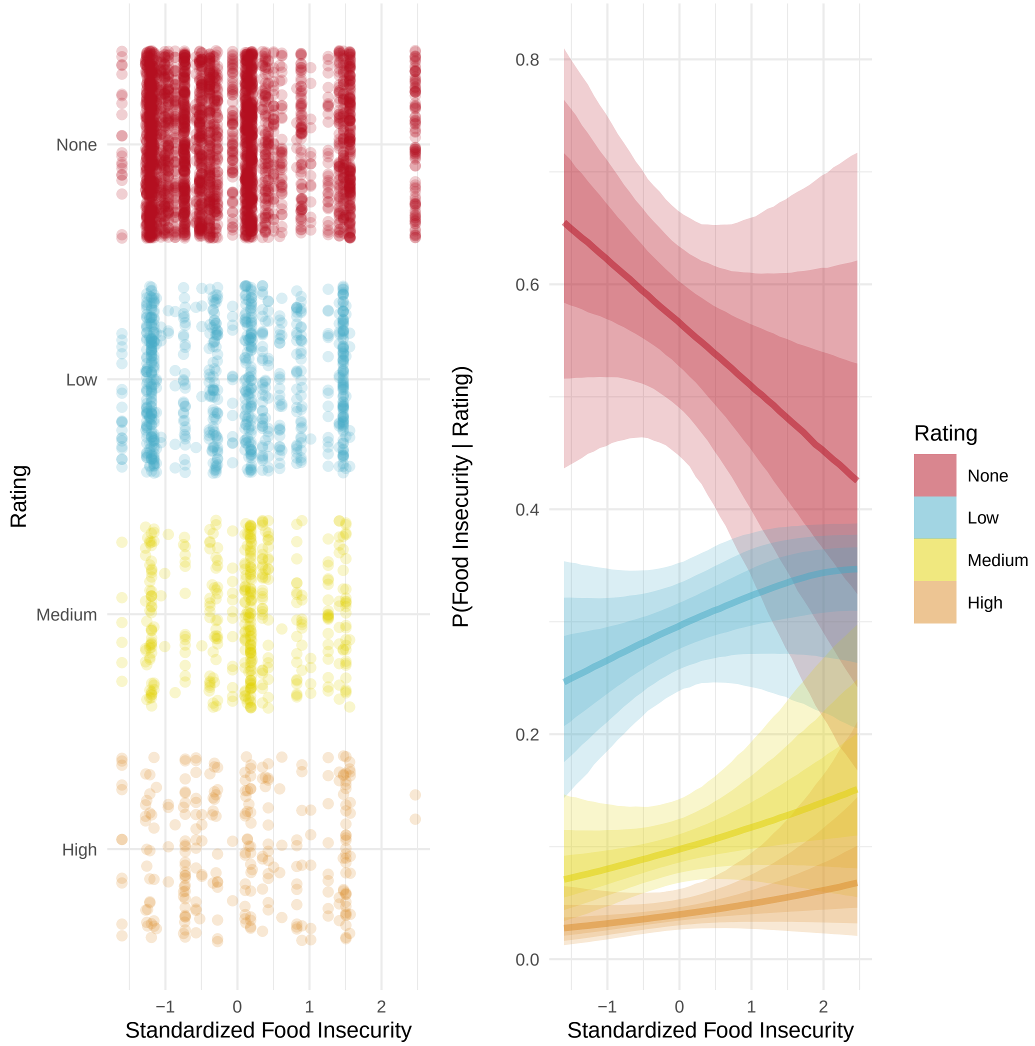

Posterior predictions for resource scarcity and predictors on female preference from Model 5

Posterior predictions of fixed effects parameters for model 5, which includes rater age, age difference between rater and ratee, number of dependents, food insecurity, and an interaction parameter between food insecurity and number of dependents. Raw data on left plots shows jittered responses by predictor. Ordinal predictions in plot on the right show predictions for the probability of each individual rating, with posterior median and 50%, 80%, and 95% credible intervals shown. Where interactions are shown, food insecurity is split into three categories, mean, -1 standard deviation, and +1 standard deviation, and raw data and predictions plotted.

Figure S11 - Age difference

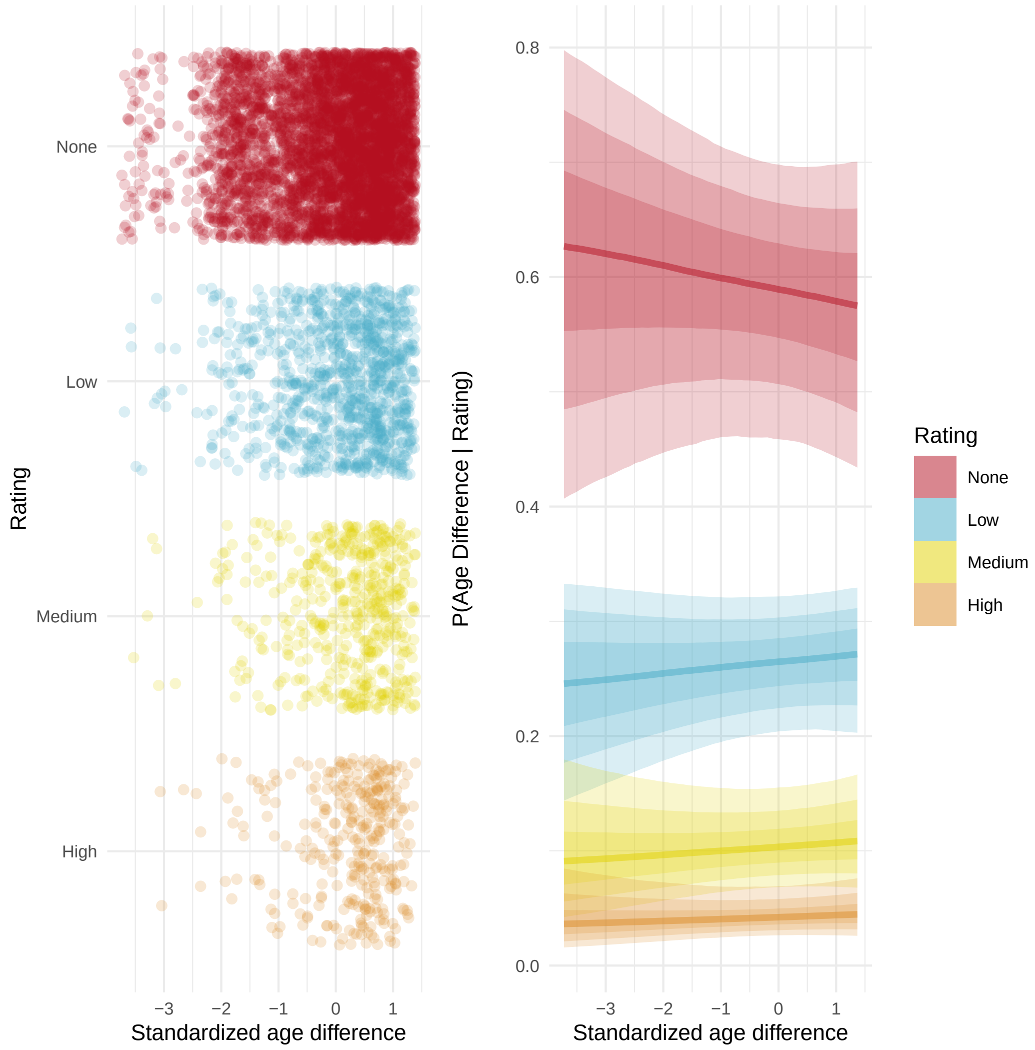

Figure S12 - Rater age

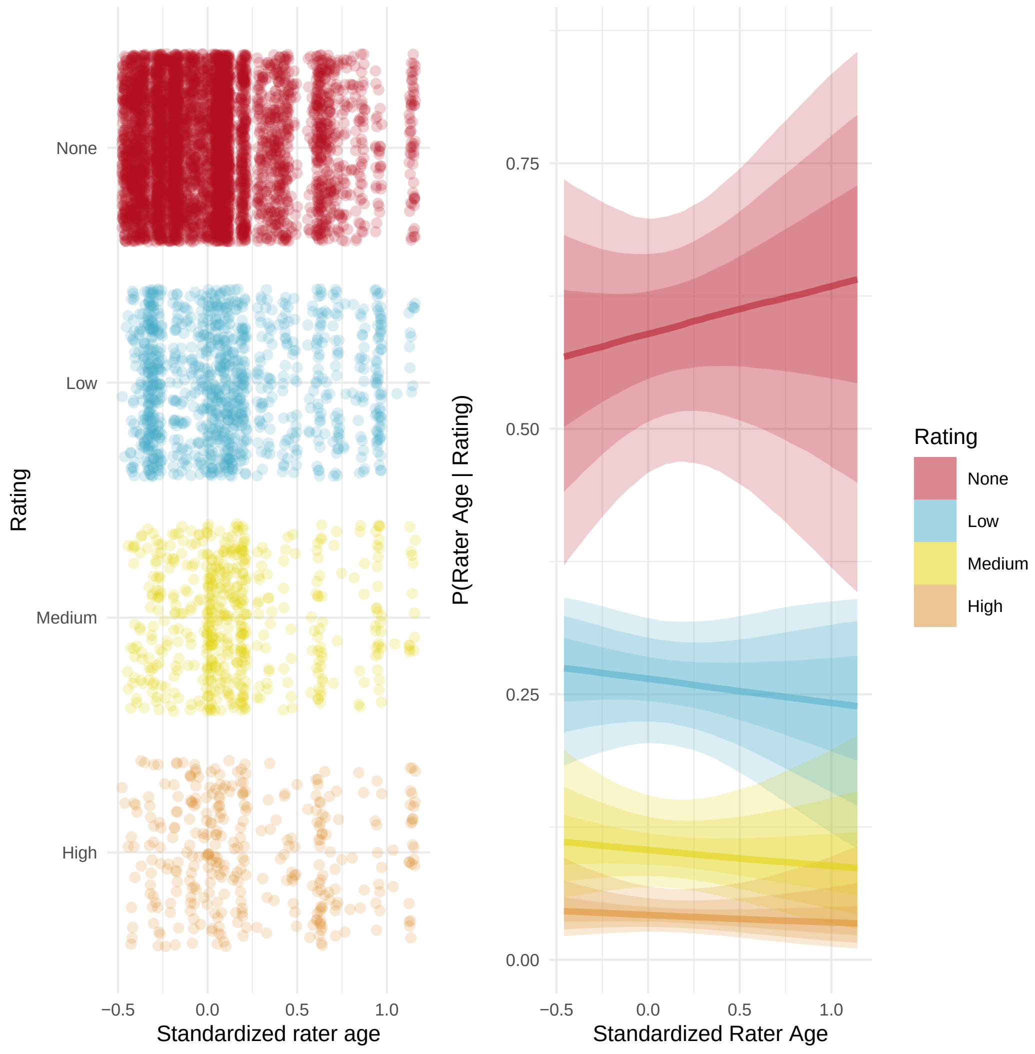

Figure S13 - Number of Dependents

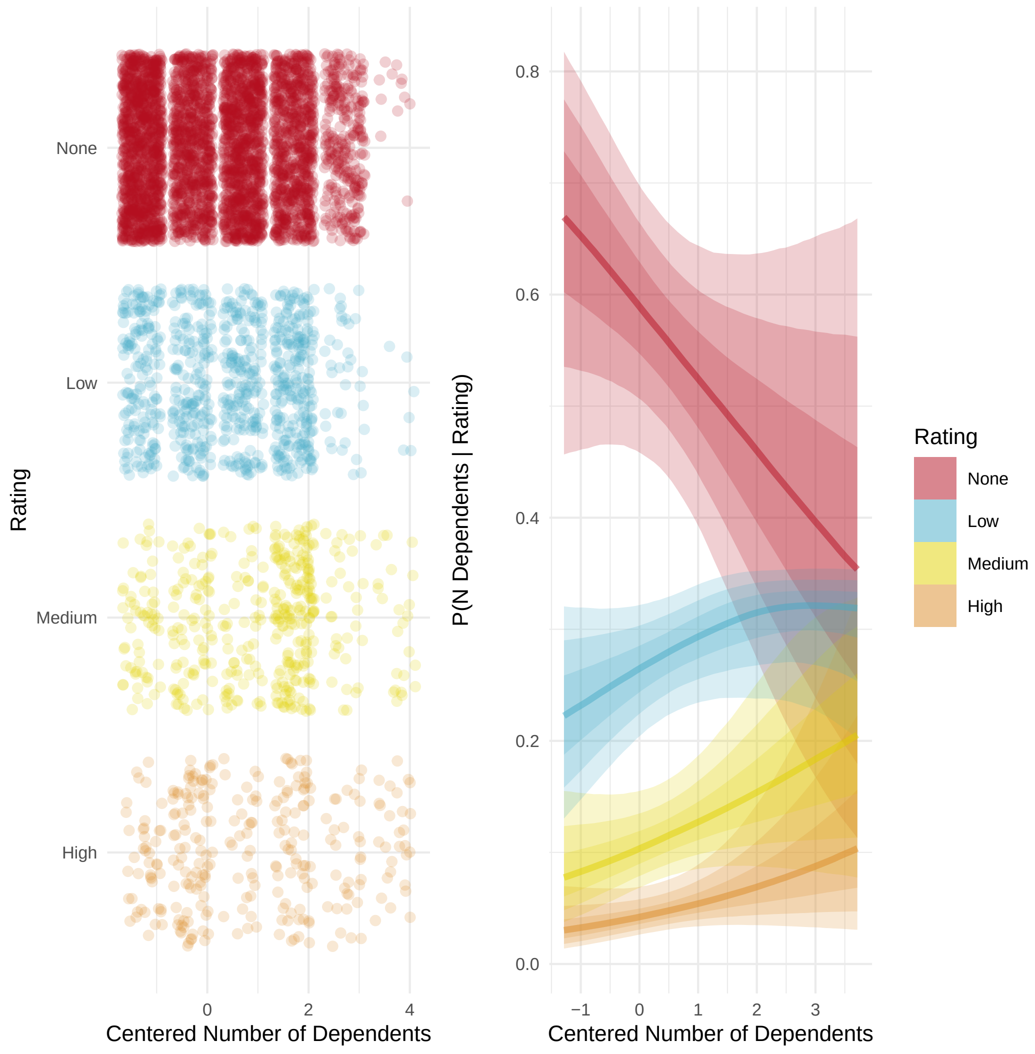

Figure S14 - Food Insecurity

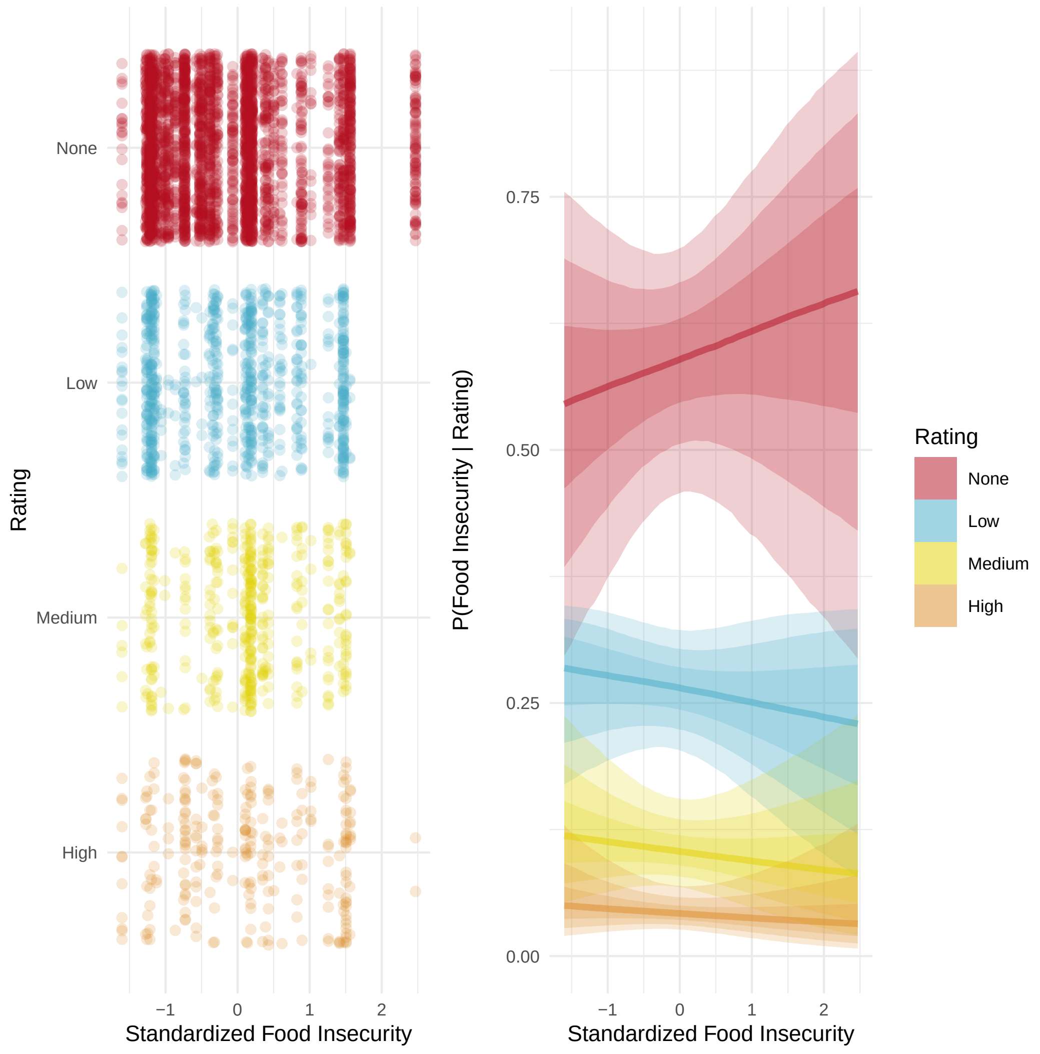

Figure S15 - Food Insecurity x Dependents

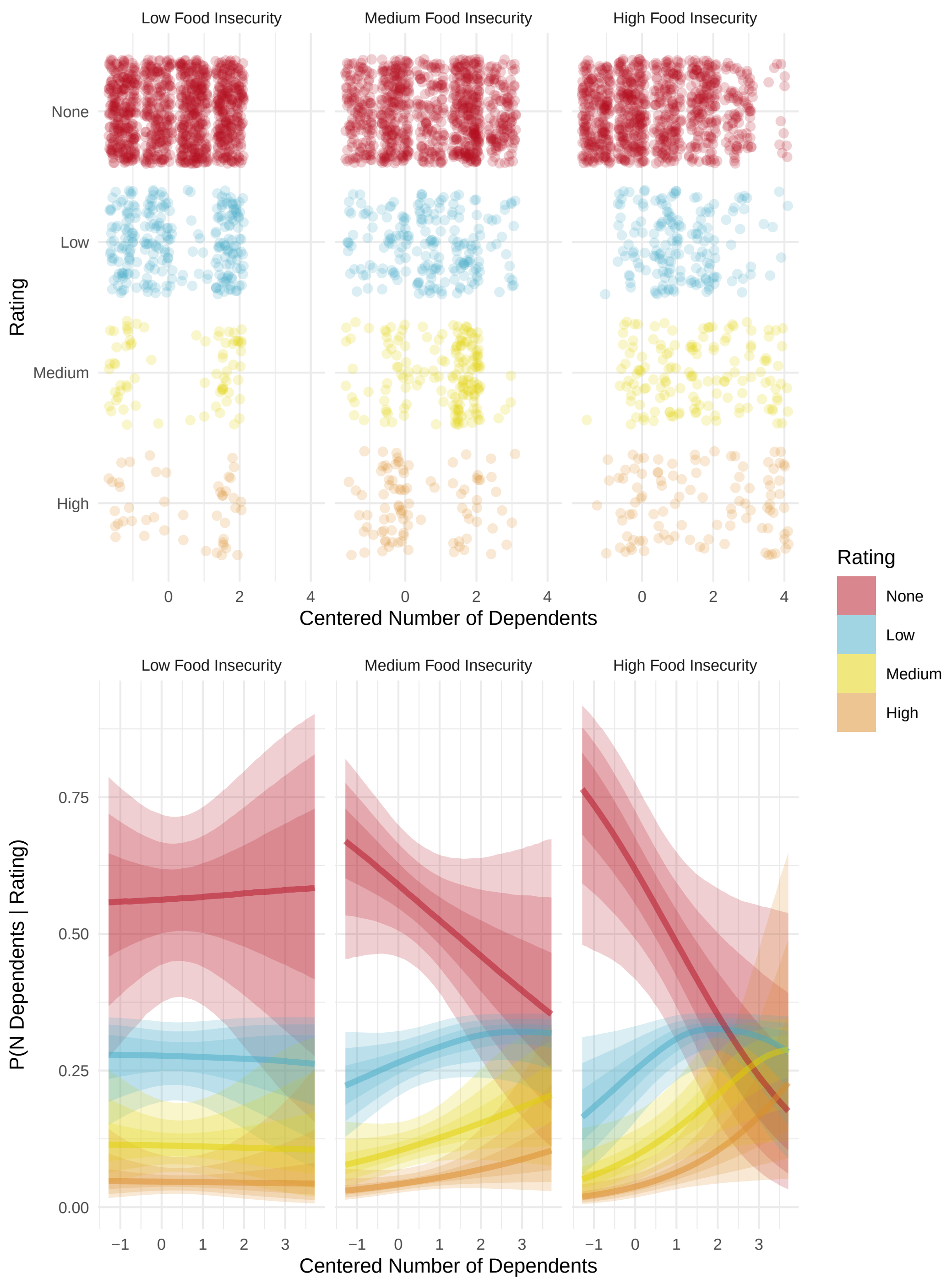

Male Wealth

Examining the impact of male wealth on female preferences. Models below modified those described previously. Interactions denote two way interactions between wealth and food insecurity and number of dependents. Untransformed posterior median and 95% credible interval shown. For interactions, trait probabilities are split into three categories, mean, -1 standard deviation, and +1 standard deviation, and predictions plotted.

Figure S16 - Wealth model coefficient comparison

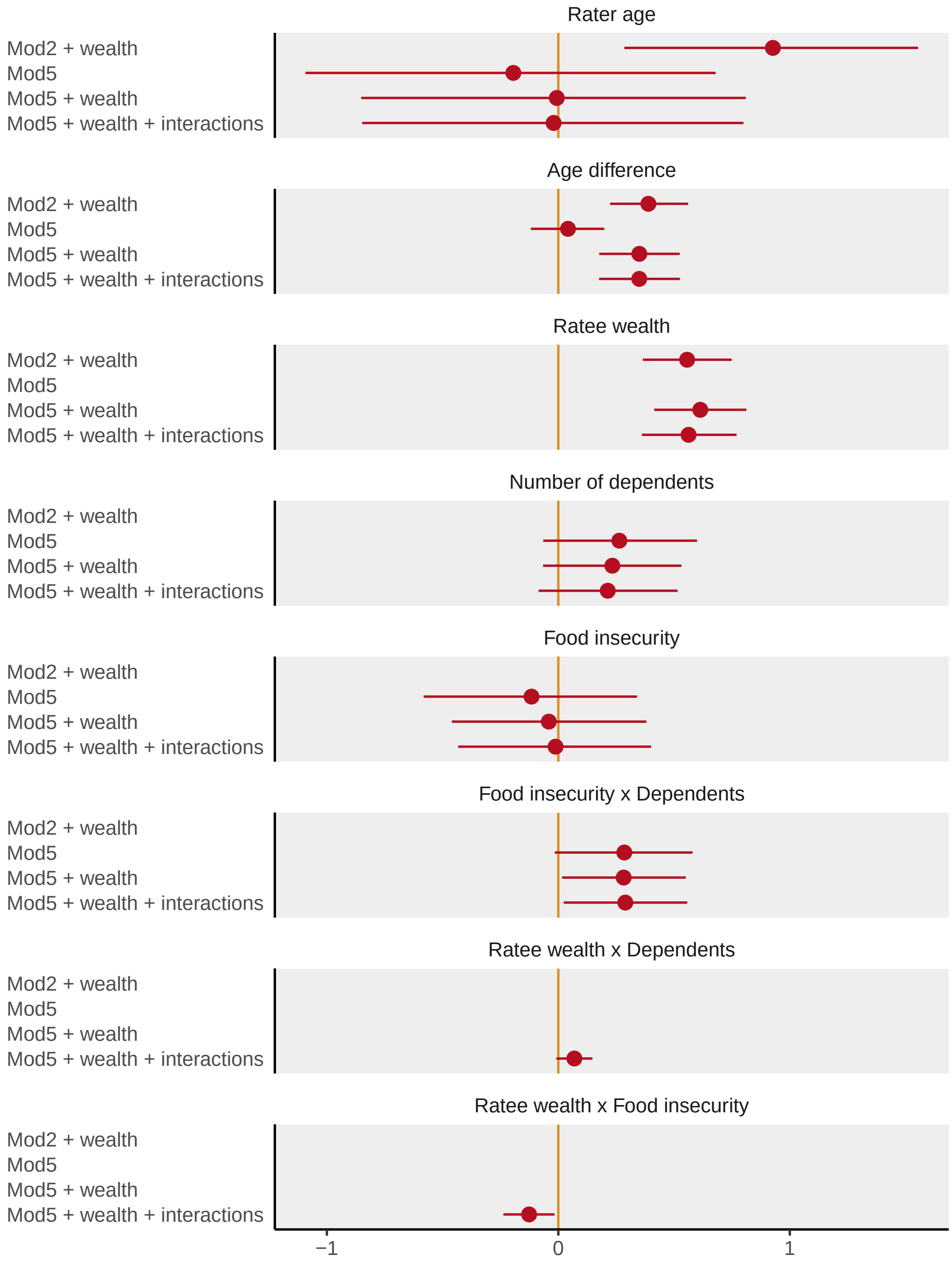

Figure S17 - Wealth

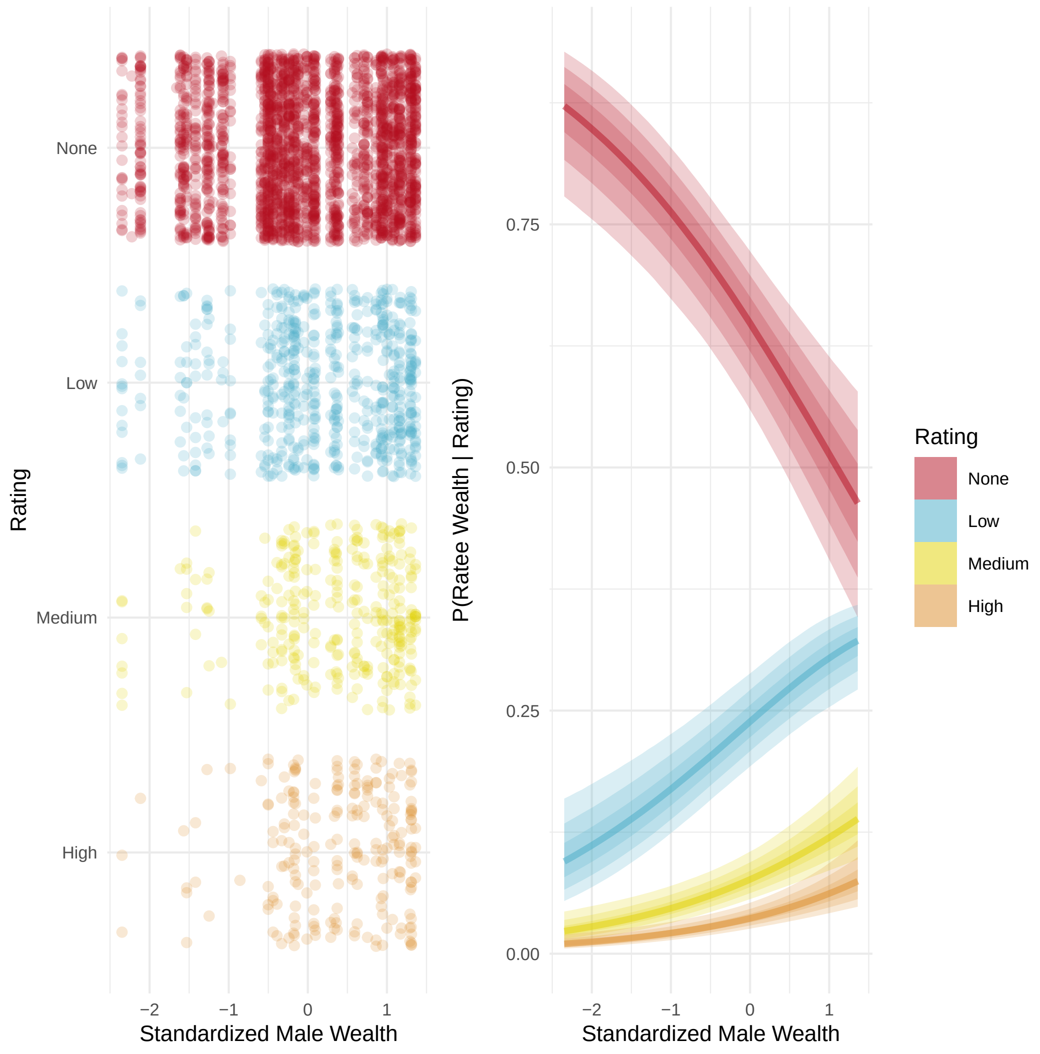

Figure S18 - Wealth x Food Insecurity and Dependents

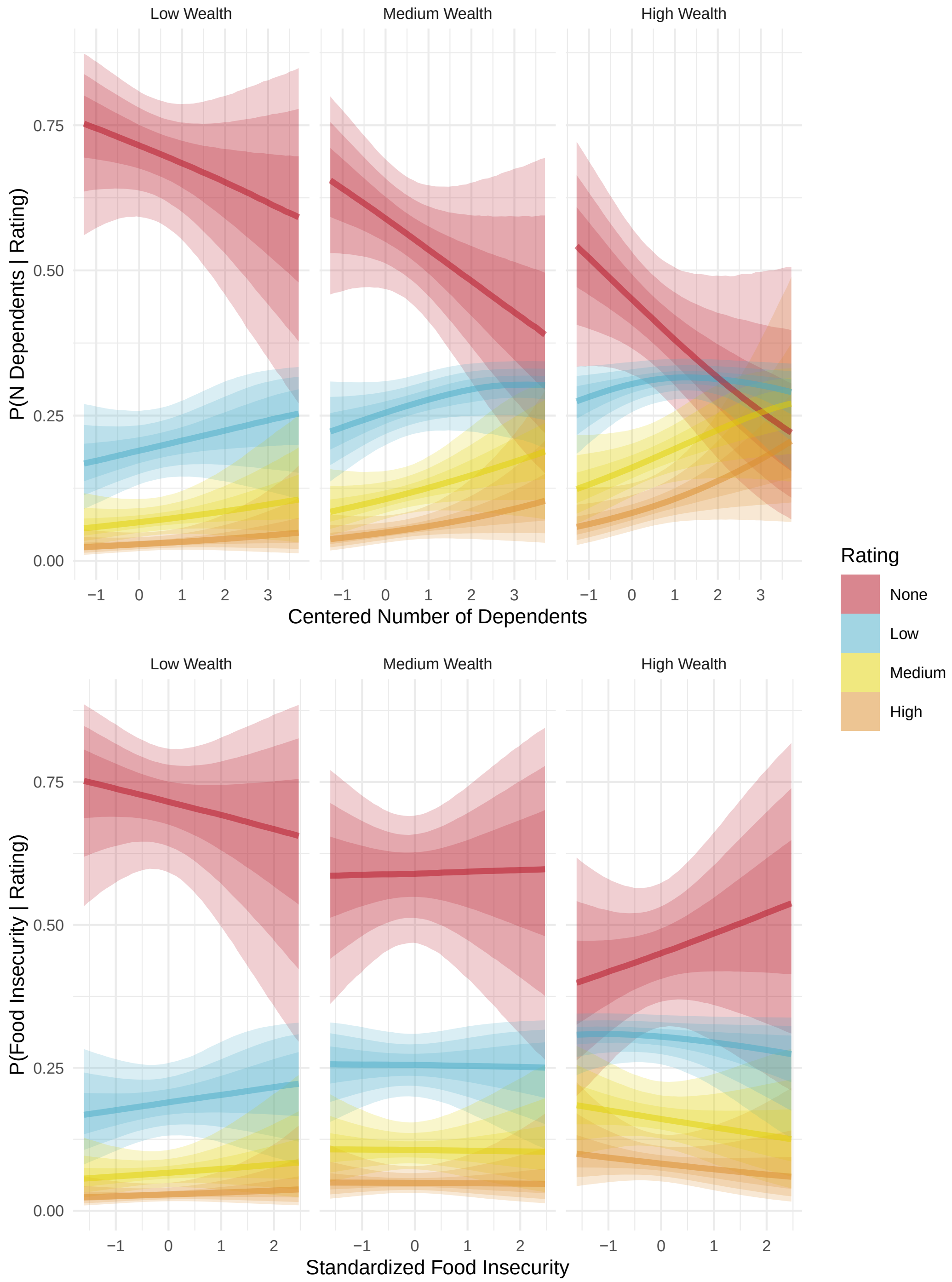

Male Traits on outcomes

Here posterior predictions for centered male traits and two-way interactions on number of dependents and food insecurity are shown. For interactions, trait probabilities are split into three categories, mean, -1 standard deviation, and +1 standard deviation, and predictions plotted.

Figure S19 - Interactions with male attractiveness

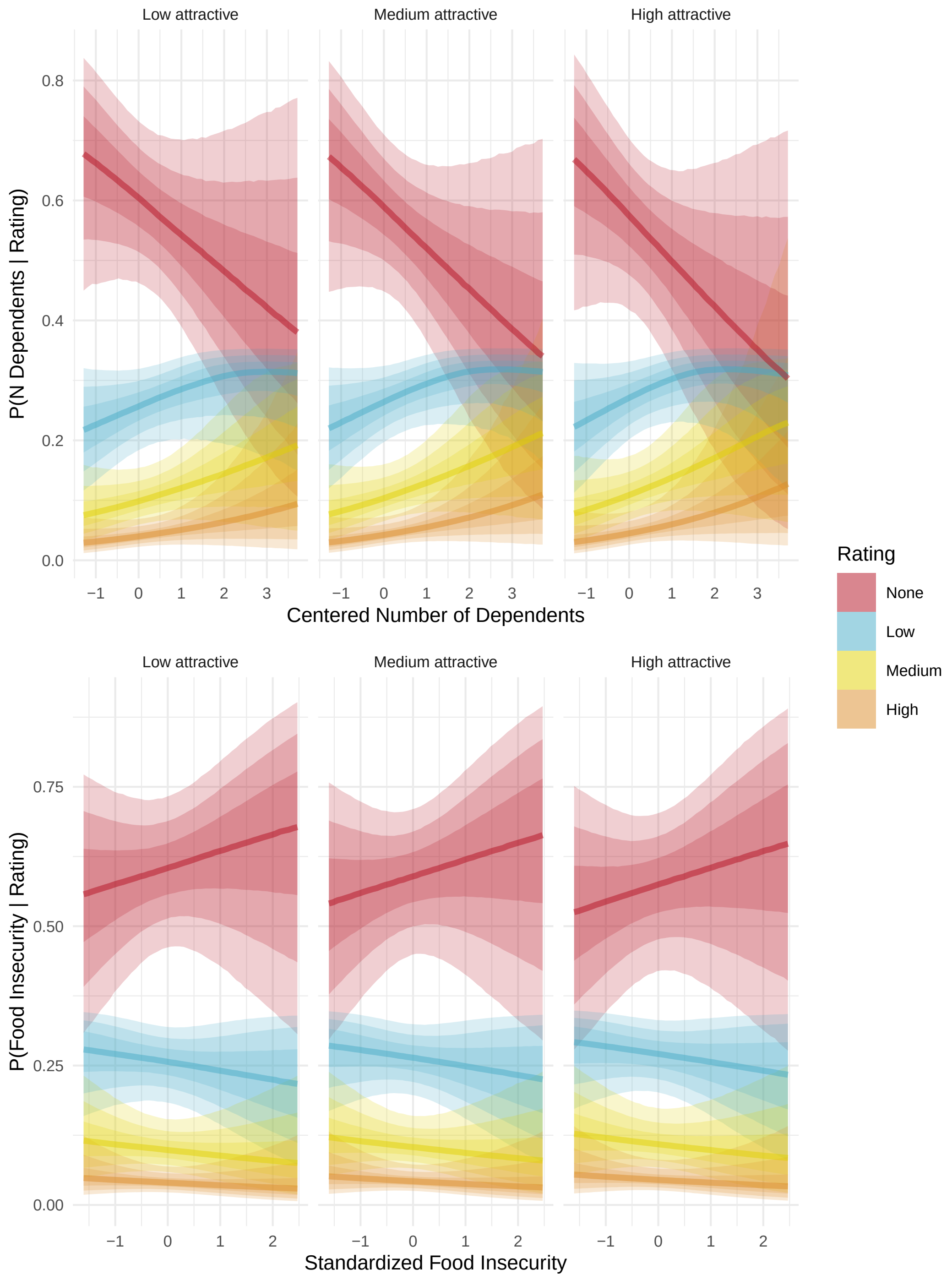

Figure S20 - Interactions with male generosity

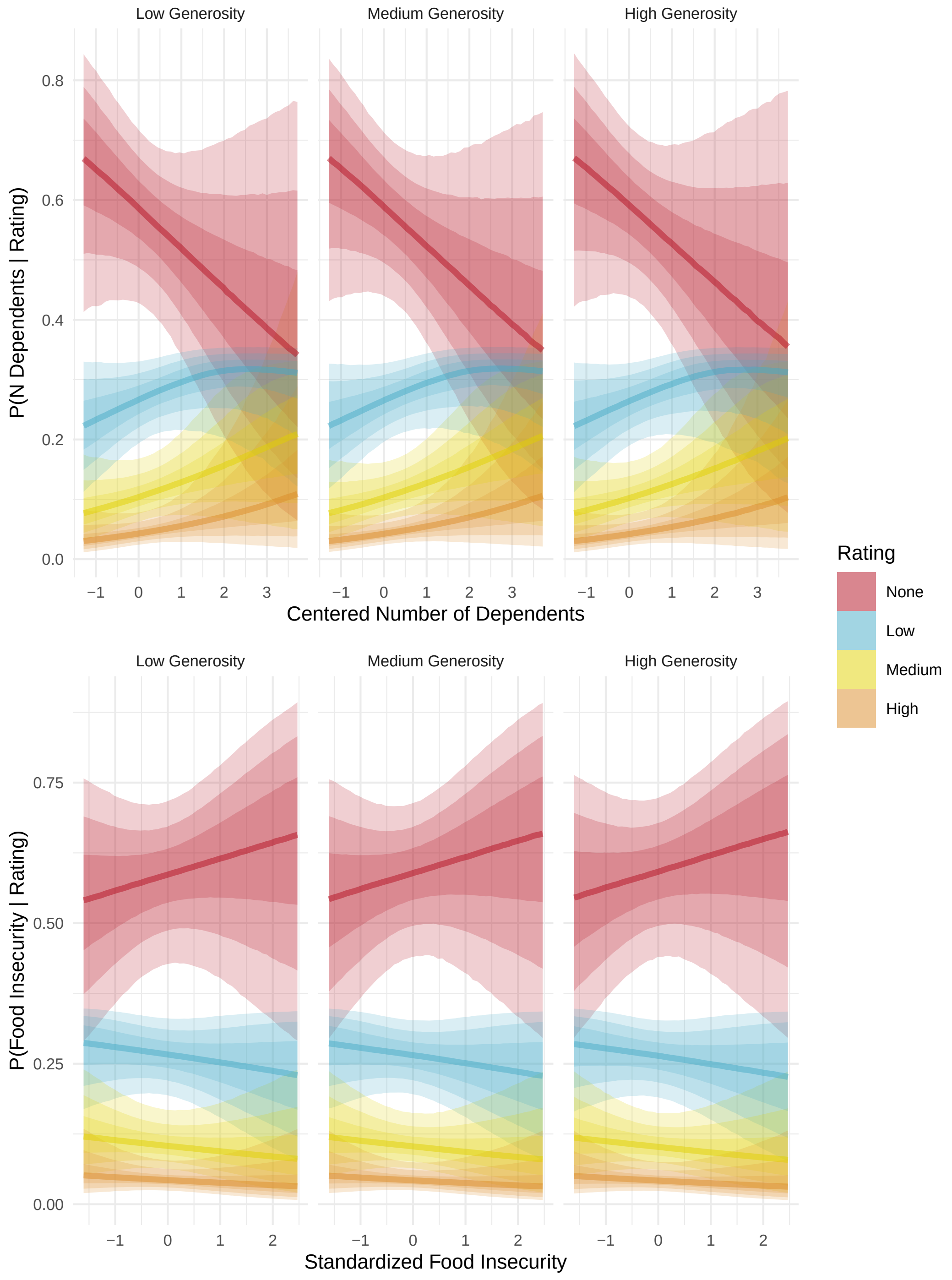

Figure S21 - Interactions with male influentialness

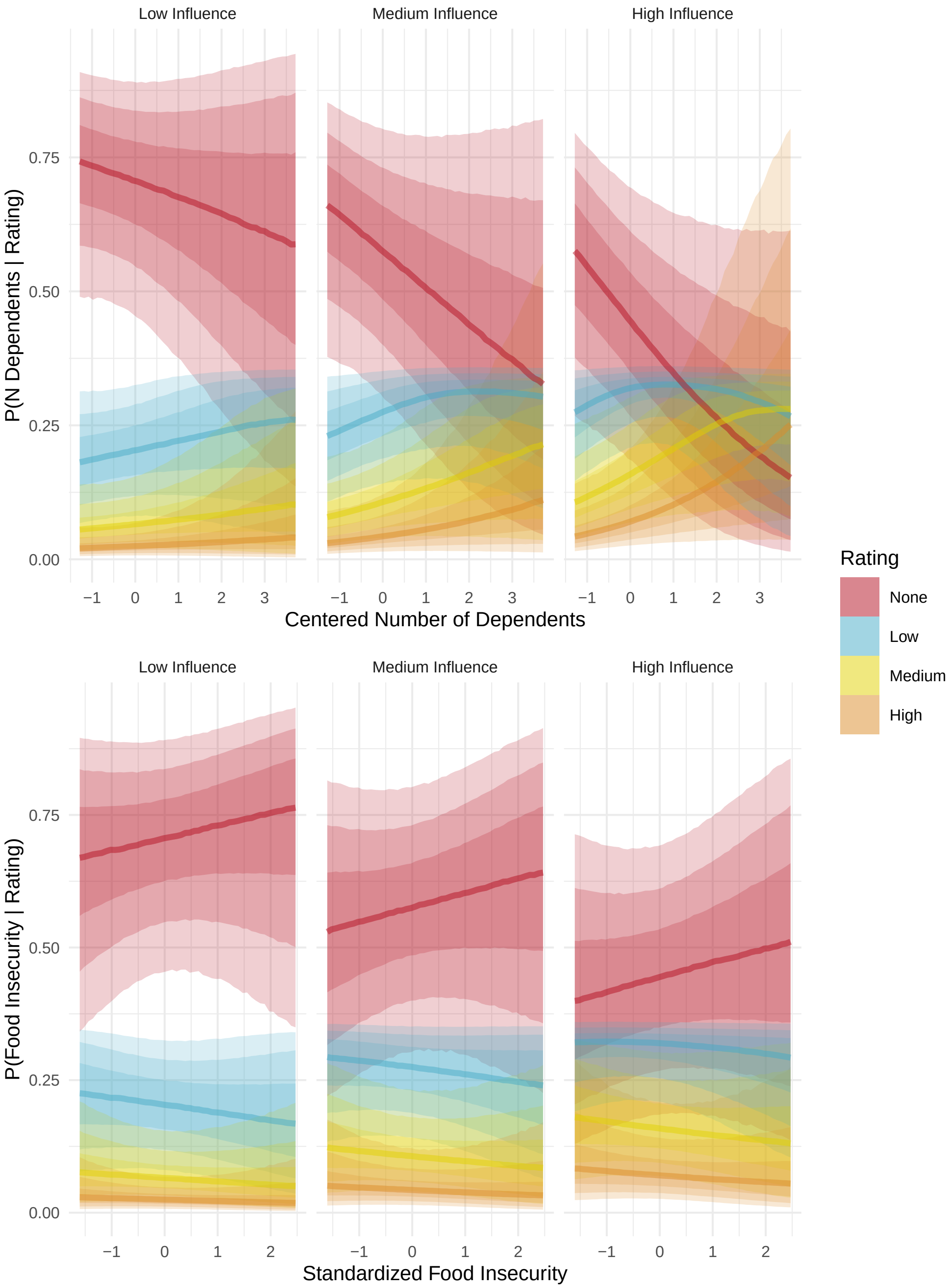

Figure S22 - Interactions with male hardworking

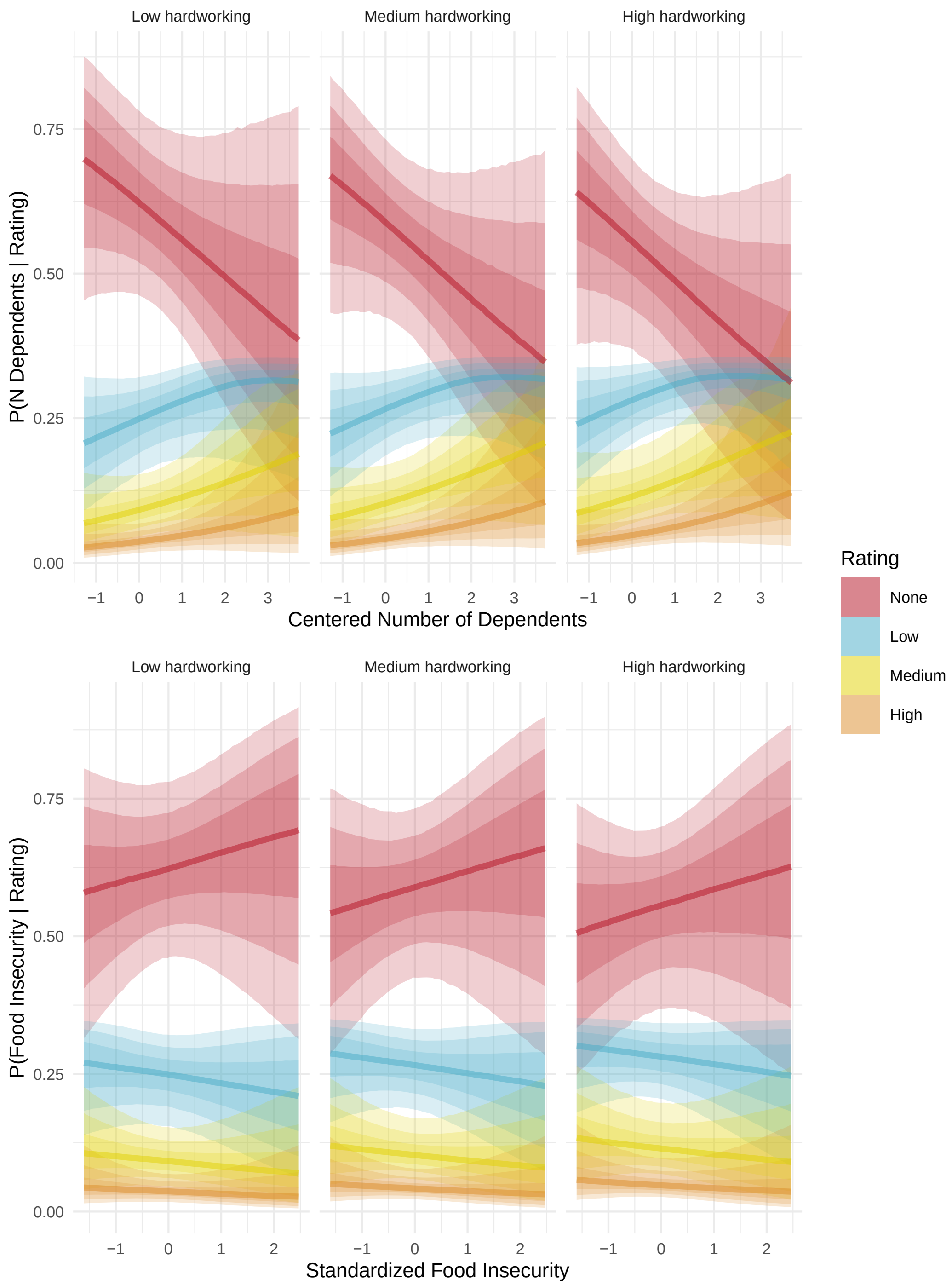

Female desirability on preference

Posterior predicts for the influence of female desirability coefficients (posterior median and standard deviation from a varying intercepts model predicting male preference of females) on female mate preferences. Model includes varying intercepts for rater and ratee, age difference, rater age, and desirability coefficient only. Note that desirability coefficient is not related to number of dependent children in this sample.

Figure S23 - Female desirability on preferences

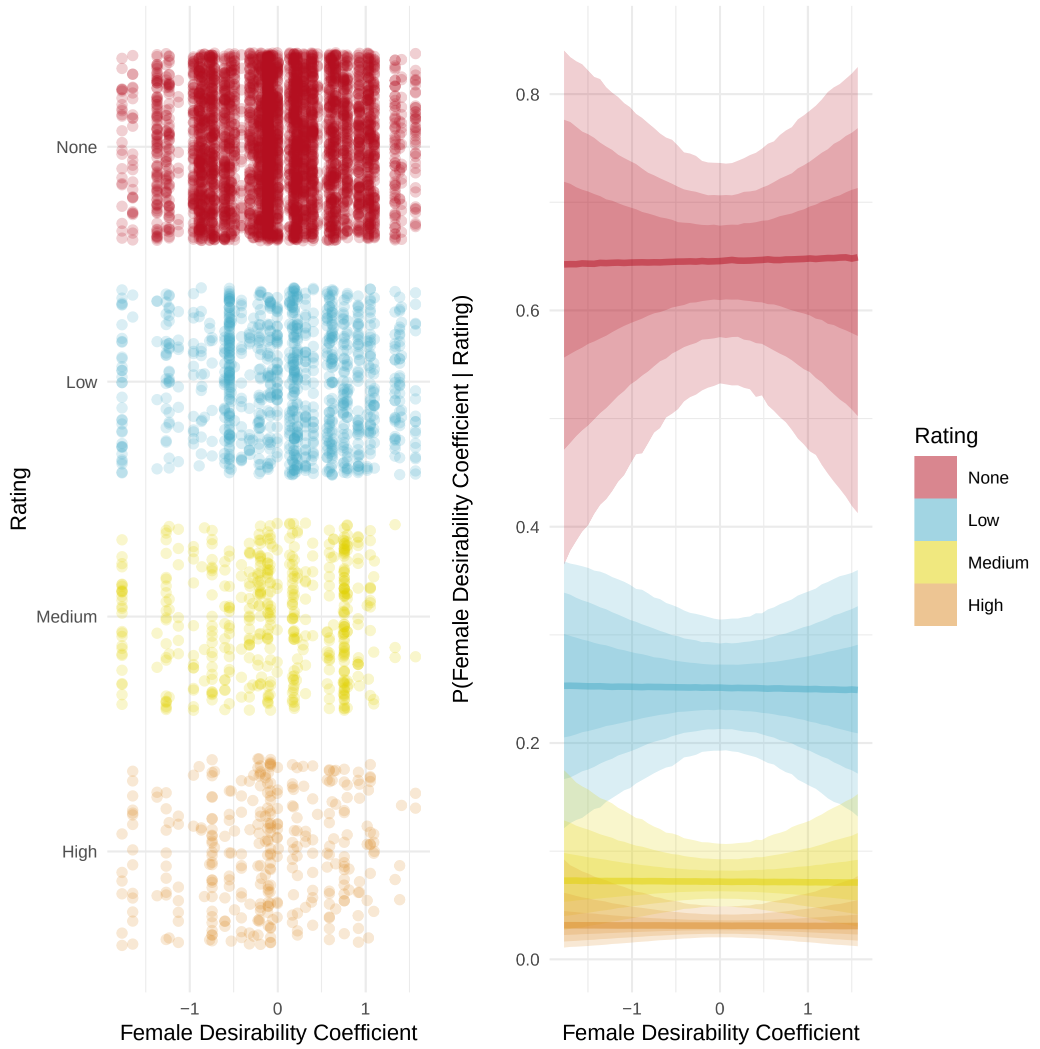

References

Bürkner, Paul-Christian. 2017. "Brms: An R Package for Bayesian Multilevel Models Using Stan." *Journal of Statistical Software* 80 (1): 1–28. https://doi.org/10.18637/jss.v080.i01.

Deitcher, Megan, Terri Ballard, Anne Swindale, and Jennifer Coates. 2010. "Validation of a Measure of Household Hunger for Cross-Cultural Use." FHI 360. Washington DC.

Gabry, j, and T Mahr. 2019. "Bayesplot: Plotting for Bayesian Models."

Kay, M. 2020. "Tidybayes: Tidy Data and Geoms for Bayesian Models."

Robinson, D, and A Hayes. 2019. "Broom: Convert Statistical Analysis Objects into Tidy Tibbles."

Team, R Core. 2019. "R: A Language and Environment for Statistical Computing." Vienna, Austria: R Foundation for Statistical Computing.

Wickham, H. 2020. "Modelr: Modelling Functions That Work with the Pipe."

Wickham, Hadley. 2017. "Tidyverse: Easily Install and Load the 'Tidyverse'."

Wilke, Claus. 2017. "Cowplot: Streamlined Plot Theme and Plot Annotations for 'Ggplot2'."
